# Ashes to eye: A skilled snake handler’s experience with ophthalmic envenomation

**DOI:** 10.1371/journal.pntd.0011264

**Published:** 2023-04-26

**Authors:** Harry F. Williams, Karin Moejes, Jarred Williams, José R. Almeida, Ravi Savania, Subramanian Senthilkumaran, Ketan Patel, Sakthivel Vaiyapuri

**Affiliations:** 1 Toxiven Biotech Private Limited, Coimbatore, Tamil Nadu, India; 2 School of Biological and Environmental Sciences, Liverpool John Moores University, Merseyside, United Kingdom; 3 School of Pharmacy, University of Reading, Reading, United Kingdom; 4 Manian Medical Centre, Erode, Tamil Nadu, India; 5 School of Biological Sciences, University of Reading, Reading, United Kingdom; Fundação de Medicina Tropical Doutor Heitor Vieira Dourado, BRAZIL

## Abstract

With the continued growth of human populations, rural urbanisation and habitat degradation are on the rise, resulting in the displacement of native wildlife and an increase in human-wildlife conflicts. The presence of human habitation and waste often attracts rodents and thereby, snakes, leading to increased snake sightings in homes. To address this problem, snake handlers, who are volunteers that remove and relocate snakes away from human development areas, are called upon. However, snake removal is a high-risk task that poses a risk of envenomation, particularly when dealing with spitting snakes. Several cobra species have the ability to spit venom. If the venom enters a person’s eye, it can result in ophthalmic envenomation, which can have serious consequences for their eyesight. Therefore, snake handlers should take precautions, wear suitable eye protection, and use appropriate tools to ensure their safety and that of the snake. In this case, an experienced snake handler was called to remove a spitting cobra, but they were ill-equipped. During the removal, the venom was sprayed across the handler’s face, and some of it entered their eye, resulting in ophthalmic envenomation. The handler promptly irrigated their eye, but medical treatment was still necessary. This report highlights the risks and consequences of ophthalmic injury and the importance of wearing appropriate eye protection and taking due care when dealing with venomous species, particularly those that can spit venom. It serves as a reminder that accidents can happen at any time and experienced snake handlers are not exempt from the risks.

## Author summary

The growth of human populations has led to an increase in rural urbanisation and habitat degradation. This, in turn, has displaced native wildlife and caused more conflicts between humans and wildlife. Human habitation and waste attract rodents and thereby, snakes, resulting in more snake sightings in homes. To deal with this issue, snake handlers/rescuers are asked to remove and relocate snakes away from human areas. However, snake removal is dangerous, especially when dealing with spitting snakes such as certain cobra species. These snakes can cause serious harm if their venom enters a person’s eye, leading to ophthalmic envenomation. Therefore, snake handlers must take precautions by wearing appropriate eye protection and using suitable tools to ensure their safety and the snake’s safety. Despite their experience, snake handlers are not exempt from the risks, as illustrated by a recent incident reported in this article where a handler was ill-equipped and suffered ophthalmic envenomation while removing a spitting cobra. This report emphasises the importance of taking due care when dealing with venomous species, particularly those that can spit venom, and wearing appropriate eye protection and using suitable tools to avoid accidents.

## 1. Introduction

In tropical and subtropical regions, human-snake conflicts are becoming increasingly worrisome as human activities such as expansion and rural development encroach on the natural habitats of snakes. The process of urbanisation is causing habitat degradation and fragmentation, leading to the displacement of herpetofauna from their preferred environments. Consequently, snakes are forced to seek new homes and attempt to coexist with humans, resulting in numerous conflicts [1–5]. Snake handlers are often called upon to remove snakes from premises and release them into areas away from human development. However, this task is not without risks, particularly when dealing with spitting cobras, as their venom can cause serious injuries if it comes into contact with the eyes [6,7]. Ophthalmic envenomation can cause intense pain, burning sensation, photophobia, hyperaemia, uveitis, and corneal erosion, which can progress to a full corneal ulcer or keratitis and the risk of corneal tearing and bacterial infection [8–10]. Therefore, it is crucial for snake handlers to use appropriate protective equipment, especially when dealing with spitting cobras. In this report, we present a case where an experienced snake handler, who is also a snakebite researcher, suffered ophthalmic envenomation because of inadequate protective gear during the removal of a spitting cobra. This report highlights the importance of using proper protective equipment when handling snakes, especially spitting cobras, regardless of the handlers’ experience and skills.

## 2. Case report

**Fig 1:**
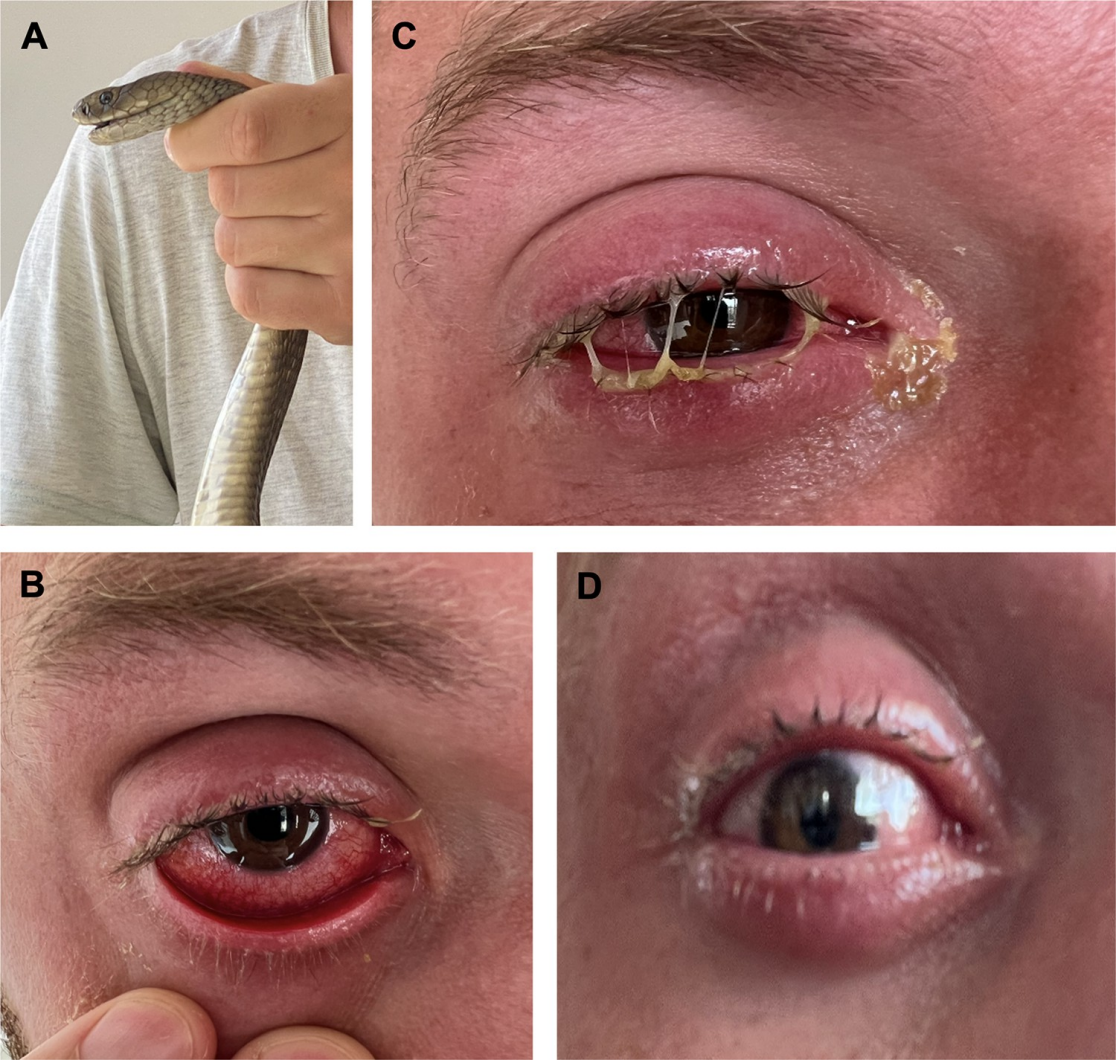
A snake handler suffers ophthalmic envenomation by an Ashe’s spitting cobra. (**A**) the offending snake was identified as an Ashe’s spitting cobra (*Naja ashei*) by a trained herpetologist and the snake handler. (**B**) the eye of the victim after the ophthalmic envenomation occurred, highlighting the irritation of the eye. (**C**) the affected eye of the victim the morning after the incident occurred, highlighting the thick mucus build-up. (**D**) the affected eye at 2 pm on the day after the incident occurred, following Diclogenta treatment.

**Fig 2:**
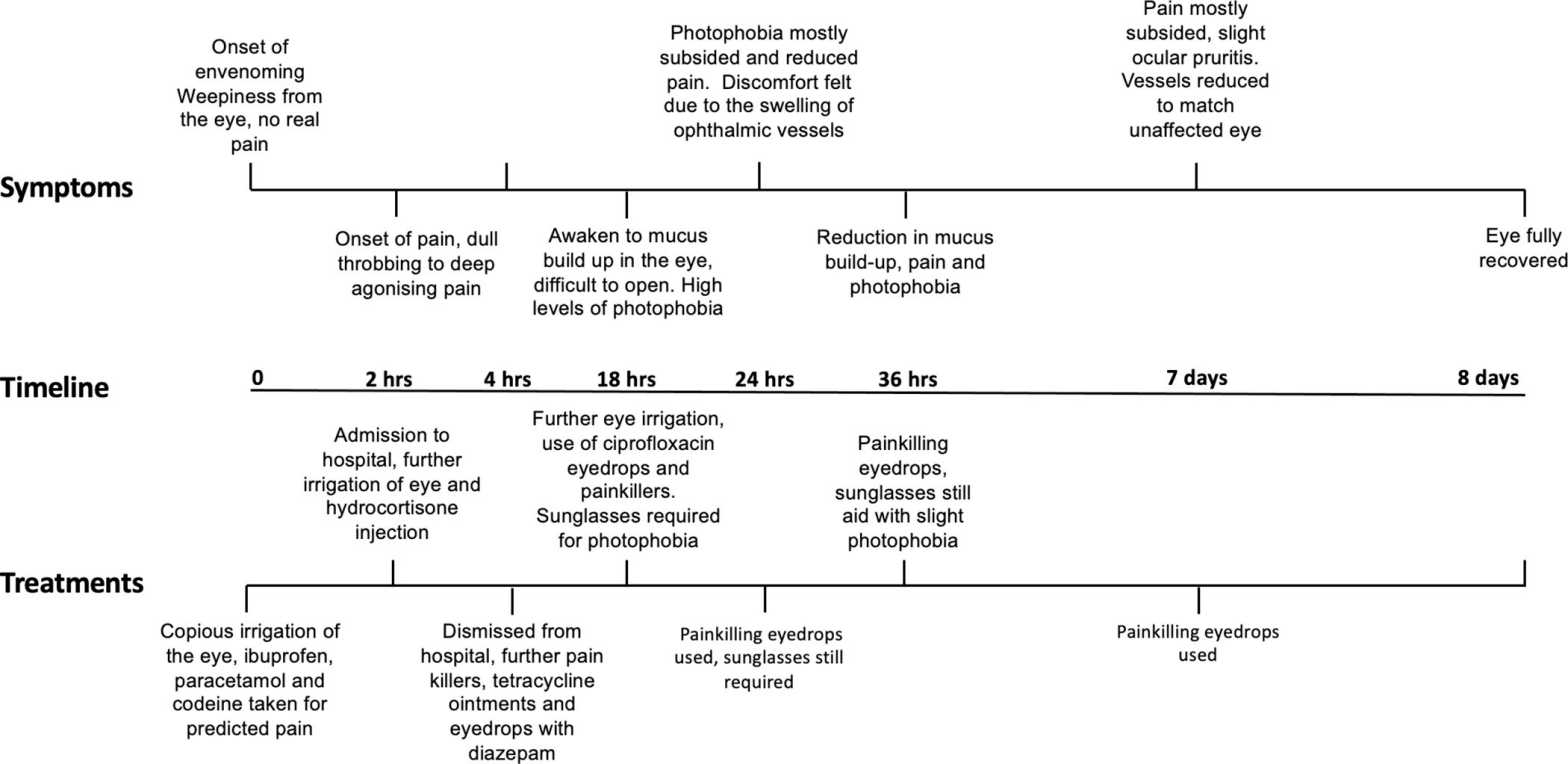
Symptoms and main events with appropriate timelines illustrating the impact and recovery following the ophthalmic envenoming by an Ashes spitting cobra.

An experienced snake handler and snakebite researcher (who is one of the authors of this article) responded to a snake rescue call on the 10^th^ of January 2023, without any protective gear due to unforeseen circumstances. The snake was identified (by the victim and a trained herpetologist) as an Ashe’s spitting cobra (*Naja ashei*) (**Fig 1A**), a venomous species known for spitting venom as a defence mechanism. Despite the risks, the handler attempted to remove the snake with a hockey stick while wearing sunglasses and turning their head away from the snake. However, the cobra spat venom across the handler’s face, which ran into their right eye, causing pain, weepiness, and photophobia that lasted for several days (**Fig 1B**). The snake was then quickly restrained, and the handler immediately rushed to a tap to flush the venom from the affected eye, but while irrigating the eye, additional venom was accidentally washed into the affected eye. To relieve the pain, the handler took one Solpadeine soluble tablet [containing paracetamol (500 mg), codeine (8 mg) and caffeine (30 mg)] as well as one ibuprofen (500 mg) tablet. A bottle of cold water was used to flush the eye and an eyewash solution of Optrex (contains aqua, extracts of *Hamemelis virginia*, alcohol, boric acid, glycerine, sodium borate & benzalkonium chloride) (around one hour after the envenomation) was also used. Approximately one hour after the ophthalmic envenoming, the pain increased significantly, and thus, another Solpadine soluble tablet was taken and the handler was admitted to a local hospital, where they received an intravenous injection of hydrocortisone (200 mg) and further irrigation of the eye with 300 mL of saline. A cotton wool swab was used in an attempt to remove any remaining venom from the eye, but this was ineffective and aggravated the eye further. No antivenom was administered in the hospital. The handler dismissed themselves from the hospital after approximately one hour of admission (i.e., two hours after the envenomation) with a prescription of tetracycline eye ointment, Acepar MR tablets (100 mg aceclofenac, 500 mg paracetamol and 375 mg chlorzoxazone) as well as a course of ciprofloxacin tablets. Two Acepar MR tablets were taken once but not ciprofloxacin, the ointment was used before taking 20 mg Diazepam to subdue the severe pain and allow the handler to sleep. Upon waking, the handler’s eye was full of thick mucus preventing the right eye from opening properly (**Fig 1C**). Most of the pain had by this point subsided. The eye was washed with saline before adding tetracycline ointment. Diclogenta (diclofenac sodium and gentamicin) and ciprofloxacin eye drops were used, and they relieved pain and irritation (**Fig 1D**). For five days following the incident, mucus regularly built up in the corner of the eye and the eye remained bloodshot for seven days. Ocular pruritis was constant from day two to day seven. Vision remained slightly blurred in the affected eye until the eight day when the quality of vision returned to that of the unaffected eye. All main events and symptomology discussed above with clear timelines are detailed in **Fig 2**.

## 3. Case discussion

With the growth of human populations and urban expansion, natural habitats are being degraded and fragmented, causing the displacement of various species including snakes [11,12]. Consequently, snakes are increasingly cohabiting with humans, leading to a rise in human-snake conflicts [2–5]. Unfortunately, this has resulted in a decline in snake populations, while the frequency of envenomations and associated deaths and disabilities has increased [13,14]. For the safe handling of snakes, handlers require comprehensive knowledge of the species present in the area, their identification, and effective handling techniques. When removing a snake, handlers must use the appropriate measures and tools to ensure the safety of the snake and humane removal, while avoiding bites and envenomations to themselves [15]. Spitting cobras such as *Naja ashei*, as described in this report, can spit venom accurately at perceived threats, causing extreme pain and sight issues in the victim [9,10]. This case report underscores the importance of proper equipment and safety measures in snake rescue operations, specifically for venom-spitting snakes.

The mechanisms through which the venom of *Naja ashei* induced ophthalmic envenomation in the handler’s eye are unclear. However, they are likely to be similar to the mechanisms reported for other elapid snakes. For example, three-finger toxins (3FTX) are key components of elapid venoms, and they are known to possess neurotoxic and cytotoxic properties that affect cell membranes [16,17]. Certain species of the *Naja* and *Hemachatus* genera contain high levels of cytotoxic 3FTXs called cardiotoxins (CTX), which are responsible for inducing ocular pain by activating sensory neurons. However, analysis of venoms from species within the *Naja* genus has shown that the abundance of CTX is comparable between spitting and non-spitting cobras, but phospholipase A_2_ (PLA_2_) levels are higher in spitters. As a result, spitting cobras induce more ocular pain than non-spitting cobras, and the evolution of spitting behaviour is closely linked to the increase in PLA_2_ levels, which enhances the analgesic effects of spitting cobra venoms [7]. Despite this, PLA_2_ alone does not typically cause visible ocular effects. In fact, it has been suggested that PLA_2_ and CTXs work synergistically to induce pain and ocular damage [18,19]. Therefore, when cobra venom toxins come into contact with the eye, they can penetrate through the corneal epithelium and bind to the stroma. Proteolytic components of the venom can also trigger the release of histamine and acetylcholine, causing pain and contributing to corneal injury, resulting in symptoms such as blurred vision, corneal oedema, conjunctival inflammation, uveitis, and bacterial infection [9,20].

The standard approach for treating ophthalmic envenomation is to irrigate the affected eye with ample amounts of neutral fluids to eliminate the venom and prevent additional damage [21]. After irrigation, the victim must seek medical attention for treatments including irrigation if necessary and receive antibacterial/antimicrobial medication to prevent infection. Topical anaesthetics have proven to be effective in reducing pain and immobilising the eye, preventing overuse, and offering relief to the affected eye [22–24]. These procedures can be recommended to all clinical settings that treat ophthalmic envenomation.

This report serves as a reminder to all snake handlers regarding the dangers associated with their job and the higher likelihood of envenomation when not adequately prepared. The report highlights that experience does not decrease the risks involved.

## Key learning points

- Increased urbanisation or deforestation results in high levels of human-snake conflicts.
- Snake handlers often work as volunteers to rescue snakes from human dwellings and release them in safe locations.
- Snake rescuing is a risky job when handling venomous snakes including snakes that can spit venoms.
- It is critical to use appropriate tools and eye protection when rescuing/handling snakes.
- This case emphasises the necessity to protect snake rescuers themselves before saving humans and snakes.

## References

[1] Dickman AJ. Complexities of conflict: the importance of considering social factors for effectively resolving human–wildlife conflict. Animal Conservation. 2010;13(5):458–66.

[2] Soulsbury CD, White PCL. Human–wildlife interactions in urban areas: a review of conflicts, benefits and opportunities. Wildlife Research. 2015;42(7):541–53, 13.

[3] Acharya KP, Paudel PK, Jnawali SR, Neupane PR, Köhl M. Can forest fragmentation and configuration work as indicators of human–wildlife conflict? Evidences from human death and injury by wildlife attacks in Nepal. Ecological Indicators. 2017;80:74–83.

[4] de Souza JC, da Silva RM, Gonçalves MPR, Jardim RJD, Markwith SH. Habitat use, ranching, and human-wildlife conflict within a fragmented landscape in the Pantanal, Brazil. Biological Conservation. 2018;217:349–57.

[5] Yue S, Timothy C., BoneBrake, Gibson L. Human-snake conflict patterns in a dense urban-forest mosaic landscape. Herpetological Conservation and Biology. 2019; 14(1):143–154.

[6] Westhoff G, Boetig M, Bleckmann H, Young BA. Target tracking during venom ‘spitting’ by cobras. Journal of Experimental Biology. 2010;213(11):1797–802. doi: 10.1242/jeb.037135 20472765 PMC2871007

[7] Kazandjian TD, Petras D, Robinson SD, van Thiel J, Greene HW, Arbuckle K, et al. Convergent evolution of pain-inducing defensive venom components in spitting cobras. Science. 2021;371(6527):386–90. doi: 10.1126/science.abb9303 33479150 PMC7610493

[8] Chu ER, Weinstein SA, White J, Warrell DA. Venom ophthalmia caused by venoms of spitting elapid and other snakes: Report of ten cases with review of epidemiology, clinical features, pathophysiology and management. Toxicon. 2010;56(3):259–72. doi: 10.1016/j.toxicon.2010.02.023 20331993

[9] Chang KC, Huang YK, Chen YW, Chen MH, Tu AT, Chen YC. Venom Ophthalmia and Ocular Complications Caused by Snake Venom. Toxins (Basel). 2020;12(9). doi: 10.3390/toxins12090576 32911777 PMC7551025

[10] Jalink MB. Ocular complications of spitting cobra venom. Indian J Ophthalmol. 2020;68(11):2632–3. doi: 10.4103/ijo.IJO_1164_20 33120721 PMC7774117

[11] Urbina-Cardona JN. Conservation of neotropical herpatofauna: research trends and challenges. Tropical Conservation Science. 2020;1(4):359–75, 17.

[12] Lehtinen RM, Ramanamanjato J-B, Raveloarison JG. Edge effects and extinction proneness in a herpetofauna from Madagascar. Biodiversity & Conservation. 2003;12(7):1357–70.

[13] Harrison RA, Hargreaves A, Wagstaff SC, Faragher B, Lalloo DG. Snake envenoming: a disease of poverty. PLoS Negl Trop Dis. 2009;3(12):e569. doi: 10.1371/journal.pntd.0000569 20027216 PMC2791200

[14] Adil S, Kanwal R., Aslam H., Ijaz S. and Afsheen S.,. Study of human impacts and interaction with herpetofauna—A review. Journal of Wildlife and Ecology. 2019;3(2):30–49.

[15] Jacobsen KS. SNAKE CONFLICT-MITIGATION IN INDIA: THE KNOWLEDGE OF THE IRULA TRIBE. Asian Affairs. 2014;45(1):108–11. doi: 10.1080/03068374.2013.826006

[16] Tasoulis T, Isbister GK. A Review and Database of Snake Venom Proteomes. Toxins (Basel). 2017;9(9). doi: 10.3390/toxins9090290 28927001 PMC5618223

[17] Ferraz CR, Arrahman A, Xie C, Casewell NR, Lewis RJ, Kool J, et al. Multifunctional Toxins in Snake Venoms and Therapeutic Implications: From Pain to Hemorrhage and Necrosis. Frontiers in Ecology and Evolution. 2019;7.

[18] Ismail M, al-Bekairi AM, el-Bedaiwy AM, Abd-el Salam MA. The ocular effects of spitting cobras: II. Evidence that cardiotoxins are responsible for the corneal opacification syndrome. J Toxicol Clin Toxicol. 1993;31(1):45–62. doi: 10.3109/15563659309000373 8433415

[19] Fung H, Choy C, Lau K, Lam T, Kam C. Ophthalmic Injuries from a Spitting Chinese Cobra. Hong Kong Journal of Emergency Medicine. 2009;16(1):26–8.

[20] Ismail M, Ellison AC. Ocular Effects of the Venom from the Spitting Cobra (Naja Nigricollis). Journal of Toxicology: Clinical Toxicology. 1986;24(3):183–202. doi: 10.3109/15563658608990457 3723645

[21] Hoffman J. Venom ophthalmia from Naja mossambica in KwaZulu Natal, South Africa: a reminder to all that for ocular chemical injury, dilution is the solution. Trop Doct. 2015;45(4):250–1. doi: 10.1177/0049475514564695 25614535

[22] Handford C. Case of venom ophthalmia following contact with Naja pallida: the red spitting cobra. J R Army Med Corps. 2018;164(2):124–6. doi: 10.1136/jramc-2017-000891 29440470

[23] Goldman DR, Seefeld AW. Ocular toxicity associated with indirect exposure to African spitting cobra venom. Wilderness Environ Med. 2010;21(2):134–6. doi: 10.1016/j.wem.2009.12.007 20591376

[24] Ang LJ, Sanjay S, Sangtam T. Ophthalmia due to spitting cobra venom in an urban setting—a report of three cases. Middle East Afr J Ophthalmol. 2014;21(3):259–61. doi: 10.4103/0974-9233.134689 25100912 PMC4123280

